# A Rare Association of Hypomagnesemia and Posterior Reversible Encephalopathy Syndrome (PRES)

**DOI:** 10.7759/cureus.41572

**Published:** 2023-07-08

**Authors:** Alexander M Ball, Appaji Rayi, Mark Gustafson

**Affiliations:** 1 Internal Medicine, Charleston Area Medical Center, Charleston, USA; 2 Neurology, Charleston Area Medical Center, Charleston, USA; 3 Emergency Medicine, Charleston Area Medical Center, Charleston, USA

**Keywords:** reversible posterior leukoencephalopathy syndrome (rpls), posterior reversible encephalopathy syndrome (pres), diarrhea, hypomagnesemia, posterior reversible encephalopathy syndrome, pres syndrome

## Abstract

Posterior reversible encephalopathy syndrome (PRES) is a rare neurologic disorder that presents with variable symptoms and symmetrical abnormal white matter signaling most commonly of the occipital and parietal lobes on magnetic resonance imaging (MRI). PRES, also known as reversible posterior leukoencephalopathy syndrome (RPLS) is commonly associated with hypertension. Hypomagnesemia’s association with PRES has been rarely reported. Here, we report a patient with severe hypomagnesemia that presented with PRES syndrome that improved with magnesium replacement. Hypomagnesemia should be considered an underlying etiology in patients presenting with PRES syndrome and should be promptly treated. The presentation can often be concerning for acute cerebrovascular accidents with symptoms of dysarthria and upper motor neuron symptoms, such as facial droop, dysarthria, and gait instability. Differential diagnosis of PRES often includes rostral brainstem infarction, transient ischemic attack, infectious encephalopathy, and metabolic/toxic encephalopathy, which is evaluated in the description of the case. The most common presentation of RPLS/PRES includes altered mental status, drowsiness, seizure, vomiting, alterations in speech including dysarthria, and visual disturbance. The first signs noted are commonly lethargy and somnolence. In this case, the patient presented notably with initial symptoms of dysarthria of speech and facial droop, with serum hypomagnesemia in which symptoms corrected rapidly with the administration of intravenous magnesium sulfate.

## Introduction

Posterior reversible encephalopathy syndrome (PRES), also known as reversible posterior leukoencephalopathy syndrome typically presents with variable neurological symptoms, such as headache, altered mental status, visual impairment, and seizures [1]. Patients commonly display symptoms, such as dysarthria of speech, ataxia, dysdiadochokinesia, akathisia, or stroke-like symptoms. Characteristically on magnetic resonance imaging (MRI) of the brain, there are signs of vasogenic edema and symmetrical abnormal signaling almost always involving the cortical and subcortical structures [2-4]. This syndrome is most seen in the setting of hypertension. Less commonly reported etiologies include the use of immunomodulatory medications, transplant patients, starvation, infection, and autoimmune disease [1,5-6]. It is also associated with pre-eclampsia and eclampsia in the obstetric setting [1,5-6]. There are previous case reports in the medical literature of critical hypomagnesemia and the diagnosis of PRES [7-9]. Previously, there have been 11 reported cases in the literature describing PRES due to hypomagnesemia [6]. In four of those cases, hypomagnesemia developed due to short bowel syndrome following abdominal surgery [8]. In the present case, hypomagnesemia is secondary to alcohol use. Similar to this case, in previously reported cases, hypomagnesemia was appreciated and found to be the culprit inciting posterior cerebellar symptoms after ruling out other etiologies, such as paraneoplastic syndrome [8]. Here, we report a 53-year-old male with an alcohol use history who was found to have MRI evidence of PRES and critical hypomagnesemia. The patient was treated with intravenous magnesium sulfate repletion and his symptoms resolved. This case is unique because the patient presented twice in two months with the same presentation and had critically low magnesium levels on both occasions. Repeat MRI within the same day of the second presentation demonstrated complete resolution of radiologic findings for PRES. The patient did not have hypertensive urgency or emergency. Before the diagnosis of PRES, other differentials were explored including rostral brainstem infarction, transient ischemic attack, infectious encephalopathy, and metabolic/toxic encephalopathy. While hypomagnesemia has been reported with PRES, we present a case of a patient who had two presentations of PRES on different occasions that were both associated with hypomagnesemia that resolved after magnesium replacement. This case serves to further establish the association between hypomagnesemia and PRES.

## Case presentation

The patient is a 53-year-old male who presented to the hospital with slurred speech, headache, and dizziness that started three days prior. He stated that he has also had episodes of diarrhea during the week before he presented. He demonstrated issues with balance prior to presentation leading to frequent and recurrent falls at home without any obvious head trauma or loss of consciousness. He also endorsed visual changes stating he had trouble seeing intermittently. The patient's blood pressure on presentation was 136/88 mmHg with the rest of his vitals being normal. The neurological examination was unremarkable except for notable dysarthria and some trouble finding words. Due to the patient’s presentation, a broad workup was initiated. Computed tomography angiography (CTA) of the head and neck, CT perfusion study, and CT head and brain without contrast were ordered and performed which ruled out evidence of large vessel occlusion (LVO), intracranial hemorrhage, and was interpreted as “no evidence of acute intracranial process.” Acute metabolic encephalopathy was ruled out in the setting of normal ammonia level, normal renal function, negative toxicology, and negative urinary drug screen. Vascular studies of the carotids did not demonstrate stenosis or occlusion. Transthoracic echocardiography did not show aortic valve insufficiency and the left ventricular ejection fraction was preserved without diastolic dysfunction. Significant laboratory findings included a complete blood count significant for anemia at 12.2 g/dL, comprehensive metabolic panel that showed an ionized calcium level of 0.73 mmol/L, a potassium level of 3.5 mmol/L, and a magnesium level of <0.5 mg/dL (Table 1).

**Table 1:** Hematology and comprehensive metabolic panel laboratory values. ALT: alanine transaminase; AST: aspartate aminotransferase; BUN: blood urea nitrogen; TSH: thyroid-stimulating hormone

Variables		Values
Hematology (g/dL)	Hemoglobin	12.2
	Hematocrit	38.5
	Platelets	284
	Leukocytes	8.5
Chemistries (mg/dL)	Sodium	142
	Potassium	3.5
	Chloride	104
	Bicarbonate	28
	BUN	7
	Creatinine	0.9
	Glucose	97
	TSH	1.594
	Alkaline phosphatase	39
	ALT	18
	AST	17
	Ammonia	28
	Urinary drug screen	Negative

**Figure 1:**
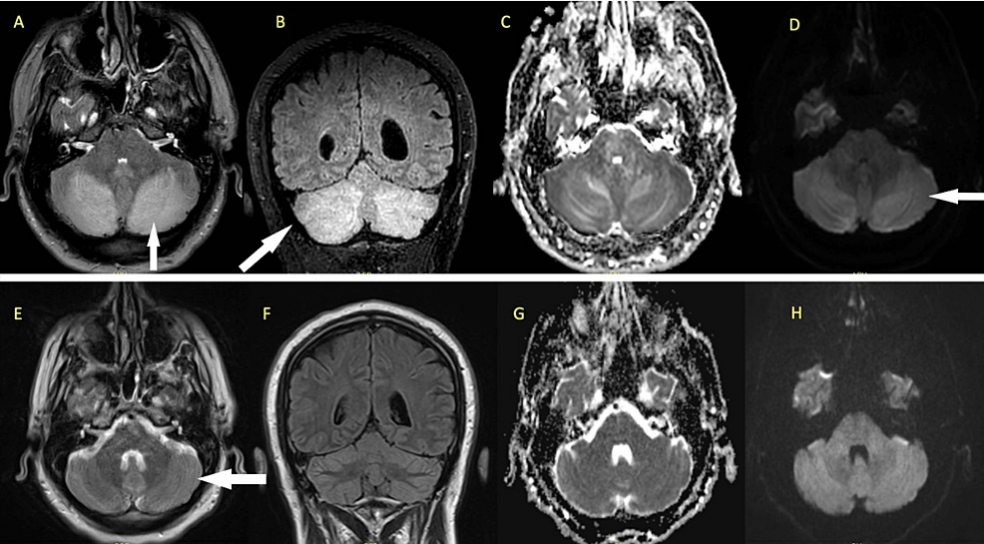
MRI brain with hyperintensities noted in the cerebellum. MRI brain images of the patient during the two hospitalizations. The upper row images are taken during the initial presentation and the images illustrate findings consistent with PRES. Arrows point to hyperintensities associated with PRES - (A) T2 axial image showing hyperintensities in the bilateral cerebellar regions, (B) coronal FLAIR images showing the findings similar to T2, (C and D) ADC and DWI axial images showing a T2 shine through artifact and no diffusion restriction ruling out concerns for stroke. Bottom-row images are taken six weeks after the previous clinical presentation, (E) T2 axial images with resolution of the previously noted cerebellar hyperintensities, (F) coronal FLAIR images with resolved cerebellar hyperintensity, and (G and H) ADC and DWI images with normal appearing cerebellum. The second presentation appeared to be clinically similar to the previous, and the patient had critically low magnesium and there were no imaging concerns for PRES. There was a complete resolution of previously visible hyperintensities within six weeks. This illustrates that the association of critically low magnesium levels with PRES is not a constant finding. PRES: posterior reversible encephalopathy syndrome; FLAIR: fluid-attenuated inversion recovery; ADC: apparent diffusion coefficient; DWI: diffusion-weighted imaging

A review of the medical record revealed that he was admitted to the hospital the previous month for similar symptoms and had an MRI that showed T2/fluid-attenuated inversion recovery (FLAIR) images with changes that were symmetric in the cerebellum consistent with PRES syndrome (Figure 1, panels A-E). During this first visit, he was admitted to the hospital and was seen by the neurology team at the time and was thought to have idiopathic PRES. Coincidentally, reviewing the medical record revealed that he had a metabolic panel that was largely similar; however, at the first visit, he had a serum potassium level of 2.9 mm/L (Table 2). At the first presentation, he also had a low serum magnesium level for which intravenous magnesium sulfate was administered. He had resolution of symptoms quickly during the first presentation. However, on discharge from the hospital, he continued drinking alcohol heavily.

**Table 2:** Comparison of critical lab values between January and February presentations with PRES. PRES: posterior reversible encephalopathy syndrome

Laboratory values	January	February
Ionized calcium (mmol/L)	0.80	0.77
Potassium level (serum) (mmol/L)	2.9	3.5
Magnesium level (serum) (mg/dL)	0.7	<0.5
Blood pressure (mmHg)	136/88	122/93

On the second visit, his symptoms were similar to the previous which raised suspicion for PRES presenting with hypomagnesemia. The patient was admitted to the hospital and evaluated by neurology. The patient’s magnesium was replaced with 4 g of IV magnesium sulfate and had resolution of his clinical symptoms and repeat brain MRI which showed resolution of the previously noted PRES findings (Figure 1, panels F-H). He was then discharged home in stable condition with a plan to follow-up with neurology as an outpatient, in which a follow-up MRI showed no findings consistent with acute thromboembolic stroke or encephalomalacia and no cerebellar intensities noted.

## Discussion

In this study, we describe an uncommon association between a critically low magnesium level causing posterior reversible encephalopathy syndrome (PRES) presenting twice with a second admission revealing resolution of the imaging findings. The other associated electrolyte disturbances (hypokalemia and hypocalcemia) in addition to the alcohol use history could also have contributed. Treatment with intravenous magnesium sulfate promptly improved his clinical symptoms suggesting this was the main underlying etiology.

Previously there has been some evidence that hypomagnesemia is associated with PRES. Chardain et al., in a retrospective study, found that all 19 patients with PRES of varying etiologies, that also had serum magnesium drawn, had hypomagnesemia in the acute phase of the disease process suggesting an association between the two [7]. In addition to this retrospective study, there have been several case reports further outlining the association between low magnesium and PRES [8,9] Additionally, other conditions that are commonly associated with hypomagnesemia have also had reports of causing cases of PRES. Fang et al. have outlined the evidence suggesting hypomagnesemia has a role in PRES associated with pre-eclampsia and eclampsia in the obstetric setting [10]. Chemotherapy and cancer treatment [11], clostridium difficile infection [12], and alcoholism have been reported as having an association with hypomagnesemia and PRES [13]. In the present case, the patient frequently drinks alcohol and was having watery diarrhea which likely contributed to serum hypomagnesemia like these previously reported cases.

The pathophysiology of PRES is unclear although the most accepted mechanisms are endothelial dysfunction and increased vascular permeability [14]. The mechanism of hypomagnesemia contributing to PRES is again unclear but its role in regulating endothelial integrity, vascular tone and reactivity, and cell membrane permeability could be reasonable justifications [14]. There have been reports that treatment of PRES with magnesium leads to improved neurologic function and recovery suggesting a need for further studies to evaluate the potential role of magnesium therapy in the treatment of PRES [15]. Here, we presented a case of a patient with diarrhea and frequent alcohol use presenting with PRES associated with hypomagnesemia that rapidly improved and had resolution of MRI findings with magnesium therapy. This case further provides evidence that magnesium therapy in the treatment of PRES needs to be further investigated.

## Conclusions

In this study, we highlighted the association between critically low magnesium levels and posterior reversible encephalopathy syndrome. Prompt recognition with an elevated level of suspicion is necessary to correlate these conditions. There is an observed rapid improvement in clinical symptoms with intravenous magnesium repletion in this patient on two separate occasions. In non-hypertensive emergency patients, other causes should be sought and treated immediately. Typically, the radiological changes will improve later in a few weeks duration after clinical recovery.
